# The clinical frailty scale, but not the FRAIL checklist is associated with mortality in old critically ill patients with COVID-19

**DOI:** 10.1186/s13054-023-04398-6

**Published:** 2023-03-10

**Authors:** Bernhard Wernly, Hans Flaatten, Susannah Leaver, Bertrand Guidet, Christian Jung, the COVIP investigators, Jesper Fjølner, Michael Beil, Sandra Oeyen, Wojtek Szczeklik, Muhammed Elhadi, Sigal Sviri, Dylan deLange, Rui Moreno, Antonio Artigas, David Dudzinski, Nicolas Serck, Helene Korvenius Nedergaard, Iman Shaat, Aliae Mohamed Hussein, Mostafa Zanaty, Ebtisam Hassanin, Aliae Mohamed Hussein, Nouralsabah Mohamed, Marwa Omar, Ghada Atef Ali Abd El-wahed, Shimaa Touny, Avinash Aujayeb, Saad Nseir, Thomas Urbina, Pierre Garcon, Jean-Philippe Rigaud, Thierry Vanderlinden, Xavier Valette, Buno Megarbane, Elodie Baron, Olivier Nigeon, Gaetan Plantefeve, Camille Foucault, Mehran Monchi, Kristina Fuest, Raphael Bruno, Malte Kelm, Hans-Joachim Kabitz, Stefan Schaller, Abdurraouf Abusalama, Hussein Embarek, Mohamed Anaiba, Ahmed Taher, Akram Alkaseek, Mirjam Evers, Willem Dieperink, Alexander Daniel Cornet, Filipa Brochado, Sonia Lopez-Cuenca, Mohammad Aldiabat, Mohammed Al-Sadawi

**Affiliations:** 1 Department of Internal Medicine, General Hospital Oberndorf, Teaching Hospital of the Paracelsus Medical University Salzburg, Salzburg, Austria; 2 Center for Public Health and Healthcare Research, Paracelsus Medical University of Salzburg, Salzburg, Austria; 3 Department of Research and Development, Haukeland University Hospital, University of Bergen, Bergen, Norway; 4 General Intensive Care, St. George’s University Hospital NHS Foundation Trust, London, UK; 5 Service de Réanimation Médicale, Hôpital Saint-Antoine, Institut Pierre-Louis d’épidémiologie et de santé publique, Hôpital Saint-Antoine, Sorbonne Université, Paris, France; 6 Division of Cardiology, Pulmonology, and Vascular Medicine, Department of Cardiology, Pulmonology and Vascular Medicine, Medical Faculty, Heinrich-Heine University Düsseldorf, Moorenstraße 5, 40225 Düsseldorf, Germany

**Dear Editor,**

Frailty is a clinical syndrome characterized by decreased reserve and resilience [1]. Identifying frailty in critically ill patients can help to guide management, including the selection of appropriate interventions and the development of care plans such as time-limited trials in patients with an unclear benefit from critical care.

The Clinical Frailty Scale (CFS) and the FRAIL checklist (1) are both tools proposed to assess frailty in older adults, but they have some key differences. The CFS is a simple, ordinal scale that assigns a score of 1 to 9 based on an assessment of the patient's level of frailty. It takes into account various physical and functional characteristics. It is quick and easy to use, and it has been validated in multiple settings [2–4]. The FRAIL checklist assesses five domains of frailty: functional impairment, recurrent hospitalizations, advanced malignancy and chronic diseases, irreversible organ failure, and long hospital stay. Patients with one of these criteria were postulated to benefit from upfront discussions about limitations of care. The FRAIL checklist has recently been proposed as a screening tool for frailty in critically ill patients [5]. Patients with CFS > 4 and FRAIL > 0 are considered vulnerable and frail.

This study aimed to compare the FRAIL and the CFS in critically ill patients with COVID-19 aged 70 years and older by incorporating the new FRAIL checklist into the protocol of the COVIP study as described in Critical Care [2]. A total of 320 patients (median age 78 ± 6 years; 39% female; median SOFA score 5 ± 3, 3-month mortality 57%) were prospectively included in the new recruitment period of the COVIP study, with 31% (n = 99) having a FRAIL > 0 and 57% (n = 136) having a CFS > 4.

The FRAIL and the CFS correlated with each other (Spearman’s rho 0.53; *p* < 0.001). Both the CFS (HR 1.14; 95% CI 1.04–1.24; *p* = 0.004) and FRAIL (1.21 95% CI 1.08–1.35; *p* = 0.001) were associated with 3-month-mortality in the univariate analysis analyzed as continuous variables. Frail patients defined by both CFS > 4 (HR 2.01 95% CI 1.50–2.69; *p* < 0.001; Fig. 1A) and FRAIL > 0 (HR 1.46; 95% CI 1.04–2.03; *p* = 0.03; Fig. 1B) evidenced worse outcomes. However, after adjustment for age, gender, SOFA and the decision to withdraw/withhold treatment during the ICU stay, CFS > 4 (aHR 1.80 95% CI 1.29–2.53; *p* = 0.001) but not FRAIL > 0 (aHR 1.16; 95% CI 0.83–1.63; *p* = 0.39) remained associated with 3-month-mortality.

**Fig. 1:**
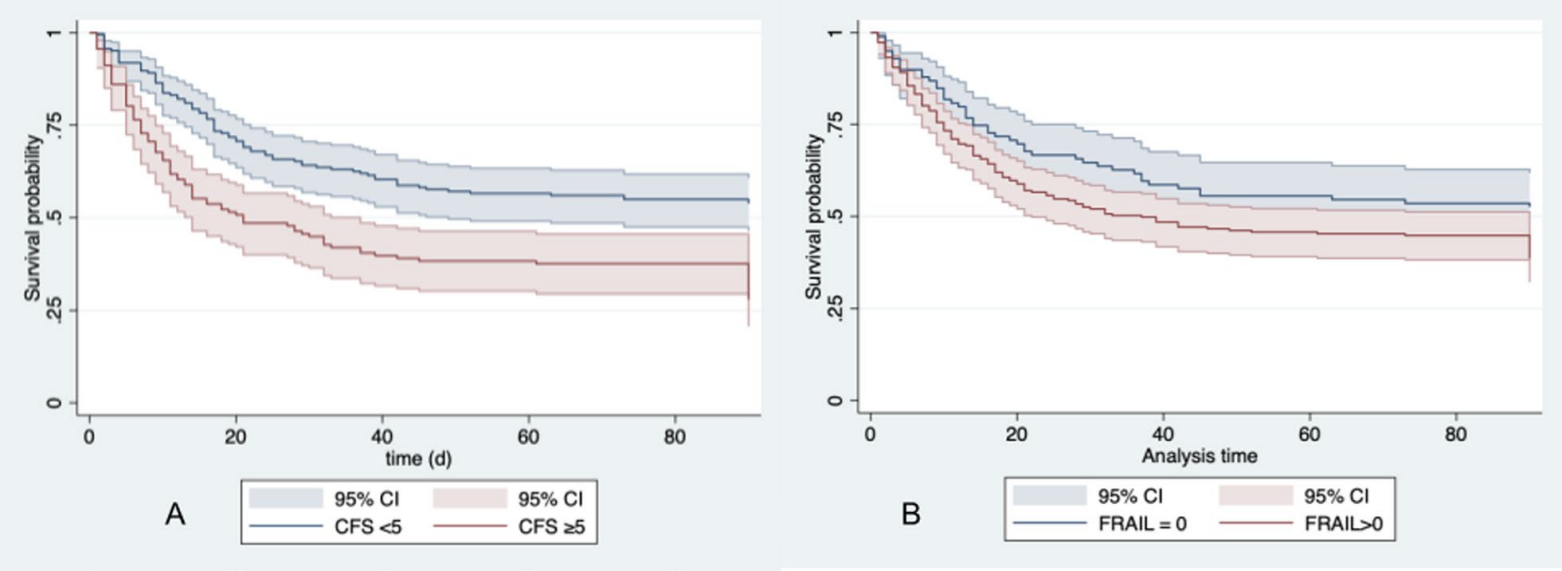
Frail patients defined by both CFS > 4 (HR 2.01 95% CI 1.50–2.69; *p* < 0.001; **A** and FRAIL > 0 (HR 1.46; 95% CI 1.04–2.03; *p* = 0.03; **B** evidenced worse outcomes

In summary, frailty is an important predictor of outcome in critically ill patients, regardless of the tool used to assess it. The FRAIL checklist identifies patients who will benefit from a time-limited trial, however, the ability to predict mortality is inherent in any critically ill patient evaluation tool. The CFS but not the FRAIL checklist was independently associated with mortality in old ICU patients. Therefore, we believe that in elderly ICU patients, CFS should be used to assess frailty because it also provides prognostic information.

## Data Availability

The datasets analyzed during the current study are not publicly available due to contractual restrictions but are available from the corresponding author on reasonable request.
